# *In Vivo* Reprogramming for Brain and Spinal Cord Repair

**DOI:** 10.1523/ENEURO.0106-15.2015

**Published:** 2015-11-09

**Authors:** Gong Chen, Marius Wernig, Benedikt Berninger, Masato Nakafuku, Malin Parmar, Chun-Li Zhang

**Affiliations:** 1 Department of Biology, Huck Institutes of Life Sciences, The Pennsylvania State University, University Park, Pennsylvania 16802; 2 Institute for Stem Cell Biology and Regenerative Medicine, Stanford, California 94305; 3 Institute of Physiological Chemistry, University Medical Center Johannes Gutenberg University Mainz, 55128 Mainz, Germany; 4 Division of Developmental Biology, Cincinnati Children's Hospital Research Foundation, and Department of Neurosurgery, University of Cincinnati College of Medicine, Cincinnati, Ohio 45229‐3039; 5 Division of Neurobiology and Lund Stem Cell Center, Wallenberg Neuroscience Center, Lund University, S‐221 84 Lund, Sweden; 6 Department of Molecular Biology, Hamon Center for Regenerative Science and Medicine, University of Texas Southwestern Medical Center, Dallas, Texas 75390

**Keywords:** astrocyte, brain repair, in vivo, neuron, NG2 cell, reprogramming

## Abstract

Cell reprogramming technologies have enabled the generation of various specific cell types including neurons from readily accessible patient cells, such as skin fibroblasts, providing an intriguing novel cell source for autologous cell transplantation. However, cell transplantation faces several difficult hurdles such as cell production and purification, long-term survival, and functional integration after transplantation. Recently, *in vivo* reprogramming, which makes use of endogenous cells for regeneration purpose, emerged as a new approach to circumvent cell transplantation. There has been evidence for *in vivo* reprogramming in the mouse pancreas, heart, and brain and spinal cord with various degrees of success. This mini review summarizes the latest developments presented in the first symposium on *in vivo* reprogramming glial cells into functional neurons in the brain and spinal cord, held at the 2014 annual meeting of the Society for Neuroscience in Washington, DC.

## Significance Statement

We have had the first symposium on in vivo reprogramming at the 2014 SFN meeting held at Washington DC. Our symposium attracted more than 800 people from around the world. This symposium invited world leaders in this emerging new field to present their most exciting results on in vivo reprogramming glial cells into functional neurons. This minireview discussed the latest developments on in vivo reprogramming and its potential application for brain and spinal cord repair.

## 

Cellular reprogramming has become of great interest in both basic and applied research over the last decade (Graf, 2011). Initiated by the successful nuclear transfer experiments in mammals, the quest arose for a molecular understanding of the reprogramming process (Campbell et al., 1996). In 2006, Takahashi and Yamanaka (2006) discovered that the simple combination of a few transcription factors can initiate the reprogramming toward a pluripotent state and thus essentially mimic *in vitro* what the ooplasm can accomplish in the nuclear transfer experiment. This work also reminded the field of previous work that single transcription factors can convert closely related lineages into each other, such as fibroblasts to muscle cells and B lymphocytes to macrophages (Davis et al., 1987; Xie et al., 2004).

The induced pluripotent stem cell technology opened a new avenue using transcription factors to reprogram adult skin fibroblast cells into stem cells, which can be differentiated into a variety of target cells (Takahashi et al., 2007; Yamanaka, 2009). Further studies have demonstrated direct interlineage reprogramming of fibroblast cells into a terminally differentiated cell type, such as neuronal cells, without going through the stem cell stage (Vierbuchen et al., 2010; Pang et al., 2011; Pfisterer et al., 2011). Such direct trans-differentiation technology has been tested not only in cell cultures *in vitro*, but also inside the mouse pancreas, heart, and in particular the brain and spinal cord *in vivo* (Buffo et al., 2008; Zhou et al., 2008; Qian et al., 2012; Grande et al., 2013; Niu et al., 2013; Torper et al., 2013; Guo et al., 2014; Heinrich et al., 2014; Su et al., 2014). At the 2014 annual meeting of the Society for Neuroscience in Washington DC, we had the first symposium on *in vivo* reprogramming and discussed potential applications of reprogramming glial cells into neurons for brain and spinal cord repair. This report summarizes the work in each speaker’s laboratory.

## Reprogramming fibroblast cells into induced neurons

Vierbuchen et al. (2010) demonstrated that cells can be directly reprogrammed into even distantly-related cell types. Specifically, they showed that fibroblasts (of mesodermal origin) can be directly converted into functional neurons (which are of ectodermal origin). After a systematic screen of ∼20 factors, it was found that the combination of the three factors *Ascl1*, *Brn2*, and *Myt1l* was sufficient to convert mouse fibroblasts into cells with neuronal morphology, neuronal marker expression and, most importantly, neuronal function including the ability to generate action potentials and formation of functional synapses. These cells were termed induced neuronal (iN) cells. It was further demonstrated that iN cells can also be formed from human fibroblasts when various combinations of transcription factors were used with or without microRNAs or small molecules (Ambasudhan et al., 2011; Caiazzo et al., 2011; Pang et al., 2011; Pfisterer et al., 2011; Son et al., 2011; Yoo et al., 2011; Ladewig et al., 2012).

These findings sparked great interest in the field and opened several new research avenues. For instance, patient-derived iN cells could be used to investigate pathogenetic mechanisms and reveal cellular phenotypes that could be used as proxy for disease expression and as assay for testing therapeutic interventions such as candidate or novel small molecule drugs (Ming et al., 2011). iN cells or other induced neural cell types that are of more proliferative capacity such as induced neural progenitor cells (iNPCs) or induced oligodendrocyte precursor cells (iOPCs) could also be used as cellular grafts with therapeutic intention, such as for Parkinson’s disease or myelin diseases (Han et al., 2012; Lujan et al., 2012; Thier et al., 2012; Yang et al., 2013). On the other hand, direct reprogramming could be envisioned for *in situ* conversion of non-neuronal cells into neurons. Given the complex manufacturing and regulatory hurdles of living cells as a therapeutic approach, the prospect to accomplish neural regeneration with delivery of small molecules or viruses is very attractive. As discussed in more detail, some initial and promising results have been obtained along these lines.

On a mechanistic level, it is unclear how the expression of a small group of transcription factors can accomplish such a biologically complex task of converting one defined, mature cell type into another. Such cell lineage conversions must include many different cell biological processes like cell polarization, cell-cycle changes, cytoskeletal rearrangements, membrane compartmentalization and proper distribution of ion channels, axonal transport, and synapse formation. Work has begun to map the earliest reprogramming events on the molecular level and found that one of the three main reprogramming factors *Ascl1* has pioneer factor properties, that is it can access closed chromatin in fibroblasts and enables recruitment of other transcription factors and eventual gene transcription (Wapinski et al., 2013). Presumably, a few critical secondarily induced, downstream transcription factors execute different parts of the Ascl1-induced program (Wapinski et al., 2013). Surprisingly, it was also found that the pioneer factor activity of *Ascl1* seems sufficient to induce iN cells without any other reprogramming factors or small molecule addition (Chanda et al., 2014). On the other hand, a closely related factor *Neurog2*, is incapable of converting fibroblasts alone, but very potent to generate iN cells from undifferentiated embryonic stem (ES) cells (Zhang et al., 2013). Current work is investigating the molecular features of *Ascl1* and *Neurog2* that are responsible for these dramatic functional differences.

## Cell reprogramming and adult neural stem cells

The concept of neuronal cell reprogramming has broad implications and impact not only in translational neuroscience, but also in basic neurobiology studies. In the adult mammalian brain, neural stem cells (NSCs) persist in a few restricted regions and continuously produce new neurons throughout life. When the in vivo identity of these adult NSCs was first revealed late last century, a surprising finding was that they share many features with mature astrocytes, one of the most abundant and widely distributed cell types in the adult brain (Doetsch et al., 1999). In fact, recent transcriptome studies have demonstrated a close similarity of the overall gene expression profile between astrocytes and adult NSCs (Beckervordersandforth et al., 2010; Codega et al., 2014). Nevertheless, only NSCs, but not astrocytes, exhibit the capacity of self-renewal and multilineage differentiation, the hallmark of stem cells. Although many regulators of adult NSCs have been identified in the past 2 decades, it is not yet fully understood what the core components of the stemness molecular program are that distinguish NSCs from astrocytes. M.N. and his colleagues used the *in vivo* reprogramming paradigm to address this long-unresolved issue. They recently demonstrated that the homeodomain transcription factor (TF) *Gsx2* and the basic helix-loop-helix (bHLH) transcription factor *Ascl1* play vital roles in the activation and neurogenesis in adult NSCs (López-Juárez et al., 2013; Andersen et al., 2014). They tested whether these key regulators of adult NSCs alone can confer any capacities of stem cells to non-stem astrocytes *in vivo*. Using newly developed transgenic mice in which *Gsx2* and *Ascl1* can be ectopically expressed in mature astrocytes, they found that these factors induce mature astrocytes to exhibit many features of NSCs, including sustained proliferation and neurogenesis *in vivo* and generation of self-renewing neurospheres *in vitro*. They further presented evidence that paracrine and autocrine signaling through transforming growth factor β receptors plays a role in regulating neurogenesis by Gsx2- and Ascl1-reprogrammed astrocytes. It will be interesting to investigate whether other reprogramming factors exhibit a similar capacity to covert astrocytes and other cell types into NSCs. As such, neuronal cell reprogramming has opened a new avenue of research on the mechanisms of cell type specification in the nervous system.

## *In vivo* reprogramming adult astrocytes to neural progenitors

Although neurons are frequently lost in response to injury or degeneration, astrocytes on the other hand become reactive, proliferative, and form glial scars. Reactive gliosis and glial scars are initially protective in restricting further spreading of damages but are in long-term deleterious by acting as both physical and biochemical barriers to neural regeneration (Sofroniew, 2009).

C.-L.Z. and colleagues developed a strategy to convert resident astrocytes to proliferative neural progenitors and functionally mature neurons in the adult brain and spinal cord (Niu et al., 2013, 2015; Su et al., 2014). After screening a dozen of transcriptional regulators that play critical roles in the regulation of neural stem cells, neurogenesis and cell reprogramming, Niu et al., 2013 identified that the stem cell factor *SOX2* alone is sufficient to robustly induce DCX^+^ neuroblasts in the adult mouse brain. Encouragingly, *SOX2* has been found to possess powerful reprogramming activity (Karow et al., 2012; Ring et al., 2012). Genetic lineage mappings confirmed that these induced adult neuroblasts (iANBs) indeed originate from resident astrocytes. A time course analysis showed that iANBs are progressively generated and can be identified even in the aged mouse brain. Interestingly, BrdU-incorporation and Ki67-staining, which are indicators of cell proliferation, showed that a fraction of iANBs are still dividing, a feature consistent with native neuroblasts. Resembling the cellular sequence of endogenous neurogenesis from neural stem cells, genetic lineage tracings and immunohistochemistry further demonstrate that SOX2-dependent *in vivo* reprogramming of astrocytes passes through a neural progenitor stage prior to the appearance of iANBs (Niu et al., 2015). Together, these data suggest that SOX2-driven cell fate conversion is a nonlinear process with the potential of one reprogrammed astrocytes giving rise to multiple iANBs.

Additional factors are required for iANBs to become functionally mature neurons in the adult brain. Niu et al. (2013, 2015) identified that the neurotrophic factors BDNF and noggin are sufficient to promote survival and maturation of the newly reprogrammed neurons. Moreover, the small molecule valproic acid (VPA), a clinically used drug for the treatment of epilepsy, mania, and migraine, can replace those neurotrophic factors. Electrophysiology using live brain slices from genetically traced mice showed that astrocyte-converted neurons are electrically mature and make appropriate connections within the local neuronal networks. By applying the same reprogramming strategy, Su et al. (2014) demonstrated that SOX2 can similarly convert resident astrocytes into mature neurons in the adult spinal cord post-traumatic injury. These induced neurons can make synaptic connections with local motor neurons (Su et al., 2014).

In summary, SOX2 overexpression initiates a stepwise reprogramming process that converts resident astrocytes to expandable neural progenitors, which eventually generate mature neurons in the injured adult central nervous system. This SOX2-driven, multistep reprogramming process may provide the much-needed neurons for neural regeneration after injury or degeneration.

## *In vivo* reprogramming NG2 glia into neurons

B.B. reported recent work aiming at reprogramming resident glia into neurons in the context of a highly invasive cortical injury *in vivo*. Work from his team had previously demonstrated that astrocytes can be reprogrammed into fully functional neurons *in vitro* by retrovirus-mediated expression of *Ascl1* or *Neurog2* (Berninger et al., 2007; Heinrich et al., 2010). Moreover, combined expression of *Sox2* and *Ascl1* had been found to convert pericytes isolated from the adult human brain into induced neurons (Karow et al., 2012), encouraging his team to study now the same combination of transcription factors *in vivo* (Heinrich et al., 2014). When the cerebral cortex of adult mice was subjected to a local injury caused by a stab wound, resident macroglia and microglia were found to respond with increased proliferation as described previously (Buffo et al., 2005; Simon et al., 2011). Three days after injury, these proliferating glial populations then could be targeted by retroviruses encoding a reporter gene for control, and *Sox2* or *Ascl1* for experimental manipulation. Although neither the control vector nor Ascl1 alone induced any degree of neurogenesis as assessed by the expression of doublecortin (DCX) in the lesioned tissue, Sox2 and Ascl1 and surprisingly even Sox2 alone induced substantial numbers of DCX-positive cells 7 days after virus delivery. Fate-mapping the cells that generate these new induced neurons using Sox10-iCreERT2 mice (Simon et al., 2012) revealed that the majority of the DCX-positive cells arise from proliferative NG2 glia (Heinrich et al., 2014). Patch-clamp recording of Sox2 and Ascl1-transduced cells provided evidence for electrical properties characteristic of immature neurons. This conclusion was further corroborated by the presence of low-frequency functional synaptic input in these induced neurons as revealed both by electrophysiology and by finding their processes decorated with bouton-like swellings arising from local interneurons. Although these data are consistent with a neuronal phenotype, B.B. pointed out that some of these features may be inherited from their NG2 glial ancestors (Bergles et al., 2000). Finally, he provided surprising insights into the relevance of the injury context for the conversion process. In fact, it turned out that without prior lesioning of the cerebral cortical tissue, forced expression of Sox2 failed to convert either NG2 glia or astrocytes into DCX-positive cells. In discussing the current state-of-the-art, B.B. pointed out that although the findings of his group as well as other laboratories represent a major advance in the attempt to convert resident glia into neurons *in vivo*, there is still a long way of fundamental research required prior to making this approach a viable alternative to cell transplantation.

## Reprogramming dopaminergic neurons *in vitro* and *in vivo*

Parkinson’s disease is a neurodegenerative disorder that is a particularly interesting target for stem cell-based therapies, and clinical trials have shown that effective repair can be achieved by neural transplantation. Notably, transplanted dopamine (DA) neurons, derived from the ventral mesencephalon (VM), can functionally reinnervate the denervated striatum, restore dopamine release and, at least in some PD patients, induce substantial long-term clinical improvement (Barker et al., 2013). Despite these encouraging results, work with human fetal tissue presents a number of ethical and logistical problems and therefore does not represent a realistic therapeutic option in the future. Approaches using pluripotent stem cells to replace the scarcely available fetal tissue is underway and predicted to reach clinical trials within the next 5 years (Parmar and Björklund, 2012).

With recent advances in direct *in vivo* conversion, this approach lends promise to future therapies for brain repair in Parkinson’s disease that would alleviate the need for an exogenous cell source. The vision is that instead of neural transplantation as a method for delivering therapeutic cells, new dopamine neurons could be obtained via directly converting resident glia cells into new neurons *in situ*. To date, it has been possible to convert several types of glia into neurons *in vitro* and *in vivo* using viral mediated gene delivery. Once formed, the new neurons acquire mature neuronal characteristics in a stepwise fashion, and at the same time down-regulate glia-specific genes. M.P.’s group, and others, have shown that both resident astrocytes and NG2 glia can efficiently be converted into neurons that mature, function and integrate into existing neural circuitry (Niu et al., 2013, 2015; Torper et al., 2013, 2015; Guo et al., 2014; Heinrich et al., 2014; Su et al., 2014; Liu et al., 2015). However, unlike for direct neural conversion *in vitro*, it is yet not possible to direct the formation of dopaminergic neurons via direct conversion *in vivo. In vitro*, it is possible to change the transcription factor combination used for direct neural conversion of fibroblasts and astrocytes in order to generate subtype-specific neurons. For example, *Ascl1* (*Mash1*), *Brn2a*, and *Myt1l* (ABM) yield glutamatergic neurons (Vierbuchen et al., 2010), whereas *Ascl1* (*Mash1*), *Lmx1a/b*, and *Nurr1* (ALN) results in the formation of dopaminergic neurons when converting fibroblasts and astrocytes *in vitro* (Caiazzo et al., 2011; Torper et al., 2013). In our studies *in vivo*, however, the ALN combination fails to convert resident astrocytes or NG2 glia into dopamine neurons *in vivo*, which has been published recently (Torper et al., 2015).

Thus, to harness the full potential of *in vivo* conversion for brain repair, one has to learn how to generate specific regionalized neuronal cell types of need in a particular disease, for example, dopamine neurons for Parkinson’s disease. It is also important to keep in mind that all diseases affecting the brain may not be suitable targets for brain repair via *in vivo* reprogramming due to loss of multiple cell types, diverse loss of neurons scattered in various brain regions, etc. Nevertheless, the ability to create new neurons from resident glia in the brain opens up for new, and previously unconsidered, possibilities for brain repair.

## Therapeutic potential of *in vivo* reprogramming

G.C. and colleagues have been focusing on the potential applications of *in vivo* reprogramming for brain repair. They have first used a brain-stab injury model to test whether injury-induced reactive astrocytes can be directly reprogrammed into functional neurons in the adult mouse cortex. When ectopically expressing a single bHLH neural transcription factor *NeuroD1* in reactive astrocytes at the stab injury sites, they were able to reprogram reactive astrocytes directly into functional neurons (Guo et al., 2014). This was achieved with retroviruses that only express NeuroD1 specifically in dividing reactive glial cells in the adult mouse cortex, where normal astrocytes do not divide under physiological condition. Patch-clamp recordings in cortical slices demonstrated that these NeuroD1-converted new neurons are functional, as shown by repetitive action potentials and robust synaptic events, suggesting that the newly converted neurons form functional synapses with other neurons and have successfully integrated into local circuits. Importantly, these astrocyte-converted new neurons could survive 2–8 months in the adult mouse cortex, indicating their therapeutic potential for brain repair. Besides this brain injury model, Chen’s group further tested *in vivo* reprogramming in a mouse model of Alzheimer’s disease (AD). They show that the 5xFAD mouse brain has numerous reactive astrocytes in the cortex, and injection of *NeuroD1* retrovirus into the 14-month-old AD mouse brain can still generate many functional neurons (Guo et al., 2014), suggesting that such *in vivo* reprogramming technologies could be used to regenerate functional neurons in the adult brain. Moreover, *NeuroD1* has also been used to directly reprogram cultured human astrocytes into functional neurons (Guo et al., 2014), suggesting that such glia-neuron conversion technology may indeed be potentially applicable for human brain repair. Importantly, *NeuroD1* directly converts astrocytes and NG2 cells into neurons, without inducing a transient progenitor stage, and the conversion efficiency can be as high as 90%, making it a potential candidate for therapeutic treatment.

G.C. further discussed unpublished work at the symposium, including direct conversion of NG2 glia into GABAergic neurons and chemical reprogramming of human astrocytes into functional neurons using a cocktail of small molecules.

## Concluding remarks

Although the vast majority of cell reprogramming studies are still conducted in cultured cells, *in vivo* reprogramming starts to attract attention of both stem cell biologists and translational researchers aiming for clinical applications. Compared to conventional stem cell therapies involving the *in vitro* manufacturing and transplantation of cultured cells, the approach to reprogram specific cell types *in vivo* greatly reduces the risks associated with conventional cell therapy. Already, animal studies have indicated promising potential for the *in vivo* reprogramming approach to regenerate functional neurons in injured or diseased brain and spinal cord. Several new articles have recently been published on *in vivo* reprogramming or related studies over the past several months since our first symposium held at the 2014 SFN meeting (Liu et al., 2015; Masserdotti et al., 2015; Niu et al., 2015; Raposo et al., 2015; Torper et al., 2015). Of course, this is still the proof-of-concept that *in vivo* reprogramming may be useful for brain and spinal cord repair and there are many challenges ahead. For example, it has been successful to reprogram glial cells into glutamatergic and GABAergic neurons inside the mouse brain, but reprogramming dopaminergic neurons from glial cells *in vivo* has been difficult so far. Furthermore, it will be important to assess the long-term functional effects of neural circuits after *in vivo* reprogramming. It is also necessary to investigate whether the gene delivery and reprogramming procedure is safe *in vivo* in a variety of animal models including nonhuman primates, before applying such *in vivo* reprogramming technology in clinical trials. Despite significant challenges, we hope that concerted efforts of a growing research community will tackle these problems and some day may realize these exciting therapeutic possibilities.

## Synthesis

The decision was a result of the Reviewing Editor Jun Chen and the peer reviewers coming together and discussing their recommendations until a consensus was reached. A fact-based synthesis statement explaining their decision and outlining what is needed to prepare a revision is listed below. The following reviewers agreed to reveal their identity: Zaal Kokaia, Michael Chopp

This excellent review is on an important new research topic, i.e. in vivo reprogramming glial cells into functional neurons; which has many implications in CNS repair after injury. The information contained in this article will help advance the field. Both reviewers expressed remarkable enthusiasm towards the novelty and quality of the manuscript.

## References

[B1] Ambasudhan R, Talantova M, Coleman R, Yuan X, Zhu S, Lipton SA, Ding S (2011) Direct reprogramming of adult human fibroblasts to functional neurons under defined conditions. Cell Stem Cell 9:113-118. 10.1016/j.stem.2011.07.002 21802386 PMC4567246

[B2] Andersen J, Urbán N, Achimastou A, Ito A, Simic M, Ullom K, Martynoga B, Lebel M, Göritz C, Frisén J, Nakafuku M, Guillemot F (2014) A transcriptional mechanism integrating inputs from extracellular signals to activate hippocampal stem cells. Neuron 83:1085-1097. 10.1016/j.neuron.2014.08.004 25189209 PMC4157576

[B3] Barker RA, Barrett J, Mason SL, Björklund A (2013) Fetal dopaminergic transplantation trials and the future of neural grafting in Parkinson's disease. Lancet Neurol 12:84-91. 10.1016/S1474-4422(12)70295-8 23237903

[B4] Beckervordersandforth R, Tripathi P, Ninkovic J, Bayam E, Lepier A, Stempfhuber B, Kirchhoff F, Hirrlinger J, Haslinger A, Lie DC, Beckers J, Yoder B, Irmler M, Götz M (2010) *In vivo* fate mapping and expression analysis reveals molecular hallmarks of prospectively isolated adult neural stem cells. Cell Stem Cell 7:744-758. 10.1016/j.stem.2010.11.017 21112568

[B5] Bergles DE, Roberts JD, Somogyi P, Jahr CE (2000) Glutamatergic synapses on oligodendrocyte precursor cells in the hippocampus. Nature 405:187-191. 10.1038/35012083 10821275

[B6] Berninger B, Costa MR, Koch U, Schroeder T, Sutor B, Grothe B, Götz M (2007) Functional properties of neurons derived from in vitro reprogrammed postnatal astroglia. J Neurosci 27:8654-8664. 10.1523/JNEUROSCI.1615-07.2007 17687043 PMC6672931

[B7] Buffo A, Vosko MR, Ertürk D, Hamann GF, Jucker M, Rowitch D, Götz M (2005) Expression pattern of the transcription factor Olig2 in response to brain injuries: implications for neuronal repair. Proc Nat Acad Sci U S A 102:18183-18188. 10.1073/pnas.0506535102 16330768 PMC1312388

[B8] Buffo A, Rite I, Tripathi P, Lepier A, Colak D, Horn AP, Mori T, Götz M (2008) Origin and progeny of reactive gliosis: a source of multipotent cells in the injured brain. Proc Natl Acad Sci U S A 105:3581-3586. 10.1073/pnas.0709002105 18299565 PMC2265175

[B9] Caiazzo M, Dell'Anno MT, Dvoretskova E, Lazarevic D, Taverna S, Leo D, Sotnikova TD, Menegon A, Roncaglia P, Colciago G, Russo G, Carninci P, Pezzoli G, Gainetdinov RR, Gustincich S, Dityatev A, Broccoli V (2011) Direct generation of functional dopaminergic neurons from mouse and human fibroblasts. Nature 476:224-227. 10.1038/nature10284 21725324

[B10] Campbell KH, McWhir J, Ritchie WA, Wilmut I (1996) Sheep cloned by nuclear transfer from a cultured cell line. Nature 380:64-66. 10.1038/380064a0 8598906

[B11] Chanda S, Ang CE, Davila J, Pak C, Mall M, Lee QY, Ahlenius H, Jung SW, Südhof TC, Wernig M (2014) Generation of induced neuronal cells by the single reprogramming factor ASCL1. Stem Cell Reports 3:282-296. 10.1016/j.stemcr.2014.05.020 25254342 PMC4176533

[B12] Codega P, Silva-Vargas V, Paul A, Maldonado-Soto AR, Deleo AM, Pastrana E, Doetsch F (2014) Prospective identification and purification of quiescent adult neural stem cells from their *in vivo* niche. Neuron 82:545-559. 10.1016/j.neuron.2014.02.039 24811379 PMC4360885

[B13] Davis RL, Weintraub H, Lassar AB (1987) Expression of a single transfected cDNA converts fibroblasts to myoblasts. Cell 51:987-1000. 3690668 10.1016/0092-8674(87)90585-x

[B14] Doetsch F, Caillé I, Lim DA, García-Verdugo JM, Alvarez-Buylla A (1999) Subventricular zone astrocytes are neural stem cells in the adult mammalian brain. Cell 97:703-716. 10380923 10.1016/s0092-8674(00)80783-7

[B15] Graf T (2011) Historical origins of transdifferentiation and reprogramming. Cell Stem Cell 9:504-516. 10.1016/j.stem.2011.11.012 22136926

[B16] Grande A, Sumiyoshi K, López-Juárez A, Howard J, Sakthivel B, Aronow B, Campbell K, Nakafuku M (2013) Environmental impact on direct neuronal reprogramming *in vivo* in the adult brain. Nat Commun 4:2373. 10.1038/ncomms3373 23974433 PMC3786770

[B17] Guo Z, Zhang L, Wu Z, Chen Y, Wang F, Chen G (2014) *In vivo* direct reprogramming of reactive glial cells into functional neurons after brain injury and in an Alzheimer's disease model. Cell Stem Cell 14:188-202. 10.1016/j.stem.2013.12.001 24360883 PMC3967760

[B18] Han DW, Tapia N, Hermann A, Hemmer K, Hoing S, Arauzo-Bravo MJ, Zaehres H, Wu G, Frank S, Moritz S, Greber B, Yang JH, Lee HT, Schwamborn JC, Storch A, Scholer HR (2012) Direct reprogramming of fibroblasts into neural stem cells by defined factors. Cell Stem Cell 10:465-472. 22445517 10.1016/j.stem.2012.02.021

[B19] Heinrich C, Bergami M, Gascón S, Lepier A, Viganò F, Dimou L, Sutor B, Berninger B, Götz M (2014) Sox2-mediated conversion of NG2 glia into induced neurons in the injured adult cerebral cortex. Stem Cell Reports 3:1000-1014. 10.1016/j.stemcr.2014.10.007 25458895 PMC4264057

[B20] Heinrich C, Blum R, Gascón S, Masserdotti G, Tripathi P, Sánchez R, Tiedt S, Schroeder T, Götz M, Berninger B (2010) Directing astroglia from the cerebral cortex into subtype specific functional neurons. PLoS Biol 8:e1000373. 10.1371/journal.pbio.1000373 20502524 PMC2872647

[B21] Karow M, Sánchez R, Schichor C, Masserdotti G, Ortega F, Heinrich C, Gascón S, Khan MA, Lie DC, Dellavalle A, Cossu G, Goldbrunner R, Götz M, Berninger B (2012) Reprogramming of pericyte-derived cells of the adult human brain into induced neuronal cells. Cell Stem Cell 11:471-476. 10.1016/j.stem.2012.07.007 23040476

[B22] Ladewig J, Mertens J, Kesavan J, Doerr J, Poppe D, Glaue F, Herms S, Wernet P, Kögler G, Müller FJ, Koch P, Brüstle O (2012) Small molecules enable highly efficient neuronal conversion of human fibroblasts. Nat Methods 9:575-578. 10.1038/nmeth.1972 22484851

[B23] Liu Y, Miao Q, Yuan J, Han S, Zhang P, Li S, Rao Z, Zhao W, Ye Q, Geng J, Zhang X, Cheng L (2015) Ascl1 converts dorsal midbrain astrocytes into functional neurons *in vivo*. J Neurosci 35:9336-9355. 10.1523/JNEUROSCI.3975-14.2015 26109658 PMC6605193

[B24] López-Juárez A, Howard J, Ullom K, Howard L, Grande A, Pardo A, Waclaw R, Sun YY, Yang D, Kuan CY, Campbell K, Nakafuku M (2013) Gsx2 controls region-specific activation of neural stem cells and injury-induced neurogenesis in the adult subventricular zone. Genes Dev 27:1272-1287. 10.1101/gad.217539.113 23723414 PMC3690400

[B25] Lujan E, Chanda S, Ahlenius H, Südhof TC, Wernig M (2012) Direct conversion of mouse fibroblasts to self-renewing, tripotent neural precursor cells. Proc Nat Acad Sci U S A 109:2527-2532. 10.1073/pnas.1121003109 22308465 PMC3289376

[B26] Masserdotti G, Gillotin S, Sutor B, Drechsel D, Irmler M, Jørgensen HF, Sass S, Theis FJ, Beckers J, Berninger B, Guillemot F, Götz M (2015) Transcriptional mechanisms of proneural factors and REST in regulating neuronal reprogramming of astrocytes. Cell Stem Cell 17:74-88. 10.1016/j.stem.2015.05.014 26119235 PMC4509553

[B27] Ming GL, Brüstle O, Muotri A, Studer L, Wernig M, Christian KM (2011) Cellular reprogramming: recent advances in modeling neurological diseases. J Neurosci 31:16070-16075. 10.1523/JNEUROSCI.4218-11.2011 22072658 PMC3236609

[B28] Niu W, Zang T, Zou Y, Fang S, Smith DK, Bachoo R, Zhang CL (2013) *In vivo* reprogramming of astrocytes to neuroblasts in the adult brain. Nat Cell Biol 15:1164-1175. 10.1038/ncb2843 24056302 PMC3867822

[B29] Niu W, Zang T, Smith DK, Vue TY, Zou Y, Bachoo R, Johnson JE, Zhang CL (2015) SOX2 reprograms resident astrocytes into neural progenitors in the adult brain. Stem Cell Reports 4:780-794. 10.1016/j.stemcr.2015.03.006 25921813 PMC4437485

[B30] Pang ZP, Yang N, Vierbuchen T, Ostermeier A, Fuentes DR, Yang TQ, Citri A, Sebastiano V, Marro S, Südhof TC, Wernig M (2011) Induction of human neuronal cells by defined transcription factors. Nature 476:220-223. 10.1038/nature10202 21617644 PMC3159048

[B31] Parmar M, Björklund A (2012) Generation of transplantable striatal projection neurons from human ESCs. Cell Stem Cell 10:349-350. 10.1016/j.stem.2012.03.004 22482498

[B32] Pfisterer U, Kirkeby A, Torper O, Wood J, Nelander J, Dufour A, Björklund A, Lindvall O, Jakobsson J, Parmar M (2011) Direct conversion of human fibroblasts to dopaminergic neurons. Proc Natl Acad Sci U S A 108:10343-10348. 10.1073/pnas.1105135108 21646515 PMC3121829

[B33] Qian L, Huang Y, Spencer CI, Foley A, Vedantham V, Liu L, Conway SJ, Fu JD, Srivastava D (2012) *In vivo* reprogramming of murine cardiac fibroblasts into induced cardiomyocytes. Nature 485:593-598. 10.1038/nature11044 22522929 PMC3369107

[B34] Raposo AA, Vasconcelos FF, Drechsel D, Marie C, Johnston C, Dolle D, Bithell A, Gillotin S, van den Berg DL, Ettwiller L, Flicek P, Crawford GE, Parras CM, Berninger B, Buckley NJ, Guillemot F, Castro DS (2015) Ascl1 coordinately regulates gene expression and the chromatin landscape during neurogenesis. Cell Rep. 10.1016/j.celrep.2015.02.025 PMC5383937 25753420

[B35] Ring KL, Tong LM, Balestra ME, Javier R, Andrews-Zwilling Y, Li G, Walker D, Zhang WR, Kreitzer AC, Huang Y (2012) Direct reprogramming of mouse and human fibroblasts into multipotent neural stem cells with a single factor. Cell Stem Cell 11:100-109. 10.1016/j.stem.2012.05.018 22683203 PMC3399516

[B36] Simon C, Götz M, Dimou L (2011) Progenitors in the adult cerebral cortex: cell cycle properties and regulation by physiological stimuli and injury. Glia 59:869-881. 10.1002/glia.21156 21446038

[B37] Simon C, Lickert H, Götz M, Dimou L (2012) Sox10-iCreERT2: a mouse line to inducibly trace the neural crest and oligodendrocyte lineage. Genesis 50:506-515. 10.1002/dvg.22003 22173870

[B38] Sofroniew MV (2009) Molecular dissection of reactive astrogliosis and glial scar formation. Trends Neurosci 32:638-647. 10.1016/j.tins.2009.08.002 19782411 PMC2787735

[B39] Son EY, Ichida JK, Wainger BJ, Toma JS, Rafuse VF, Woolf CJ, Eggan K (2011) Conversion of mouse and human fibroblasts into functional spinal motor neurons. Cell Stem Cell 9:205-218. 10.1016/j.stem.2011.07.014 21852222 PMC3188987

[B40] Su Z, Niu W, Liu ML, Zou Y, Zhang CL (2014) *In vivo* conversion of astrocytes to neurons in the injured adult spinal cord. Nat Commun 5:3338. 10.1038/ncomms4338 24569435 PMC3966078

[B41] Takahashi K, Yamanaka S (2006) Induction of pluripotent stem cells from mouse embryonic and adult fibroblast cultures by defined factors. Cell 126:663-676. 10.1016/j.cell.2006.07.024 16904174

[B42] Takahashi K, Tanabe K, Ohnuki M, Narita M, Ichisaka T, Tomoda K, Yamanaka S (2007) Induction of pluripotent stem cells from adult human fibroblasts by defined factors. Cell 131:861-872. 10.1016/j.cell.2007.11.019 18035408

[B43] Thier M, Worsdorfer P, Lakes YB, Gorris R, Herms S, Opitz T, Seiferling D, Quandel T, Hoffmann P, Nothen MM, Brustle O, Edenhofer F (2012) Direct conversion of fibroblasts into stably expandable neural stem cells. Cell Stem Cell 10:473-479. 22445518 10.1016/j.stem.2012.03.003

[B44] Torper O, Ottosson DR, Pereira M, Lau S, Cardoso T, Grealish S, Parmar M (2015) *In vivo* reprogramming of striatal NG2 glia into functional neurons that integrate into local host circuitry. Cell Rep 12:474-481. 10.1016/j.celrep.2015.06.040 26166567 PMC4521079

[B45] Torper O, Pfisterer U, Wolf DA, Pereira M, Lau S, Jakobsson J, Björklund A, Grealish S, Parmar M (2013) Generation of induced neurons via direct conversion *in vivo*. Proc Natl Acad Sci U S A 110:7038-7043. 10.1073/pnas.1303829110 23530235 PMC3637783

[B46] Vierbuchen T, Ostermeier A, Pang ZP, Kokubu Y, Südhof TC, Wernig M (2010) Direct conversion of fibroblasts to functional neurons by defined factors. Nature 463:1035-1041. 10.1038/nature08797 20107439 PMC2829121

[B47] Wapinski OL, Vierbuchen T, Qu K, Lee QY, Chanda S, Fuentes DR, Giresi PG, Ng YH, Marro S, Neff NF, Drechsel D, Martynoga B, Castro DS, Webb AE, Südhof TC, Brunet A, Guillemot F, Chang HY, Wernig M (2013) Hierarchical mechanisms for direct reprogramming of fibroblasts to neurons. Cell 155:621-635. 10.1016/j.cell.2013.09.028 24243019 PMC3871197

[B48] Xie H, Ye M, Feng R, Graf T (2004) Stepwise reprogramming of B cells into macrophages. Cell 117:663-676. 15163413 10.1016/s0092-8674(04)00419-2

[B49] Yamanaka S (2009) Elite and stochastic models for induced pluripotent stem cell generation. Nature 460:49-52. 10.1038/nature08180 19571877

[B50] Yang N, Zuchero JB, Ahlenius H, Marro S, Ng YH, Vierbuchen T, Hawkins JS, Geissler R, Barres BA, Wernig M (2013) Generation of oligodendroglial cells by direct lineage conversion. Nat Biotechnol 31:434-439. 23584610 10.1038/nbt.2564 PMC3677690

[B51] Yoo AS, Sun AX, Li L, Shcheglovitov A, Portmann T, Li Y, Lee-Messer C, Dolmetsch RE, Tsien RW, Crabtree GR (2011) MicroRNA-mediated conversion of human fibroblasts to neurons. Nature 476:228-231. 10.1038/nature10323 21753754 PMC3348862

[B52] Zhang Y, Pak C, Han Y, Ahlenius H, Zhang Z, Chanda S, Marro S, Patzke C, Acuna C, Covy J, Xu W, Yang N, Danko T, Chen L, Wernig M, Südhof TC (2013) Rapid single-step induction of functional neurons from human pluripotent stem cells. Neuron 78:785-798. 10.1016/j.neuron.2013.05.029 23764284 PMC3751803

[B53] Zhou Q, Brown J, Kanarek A, Rajagopal J, Melton DA (2008) *In vivo* reprogramming of adult pancreatic exocrine cells to beta-cells. Nature 455:627-632. 10.1038/nature07314 18754011 PMC9011918

